# Current research school activities and strategies for the future

**DOI:** 10.1192/j.eurpsy.2025.141

**Published:** 2025-08-26

**Authors:** I. Agartz

**Affiliations:** 1 Clinical Medicine, University of Oslo; 2 Department of Psychiatric Research, Diakonhjemmet Hospital, Oslo, Norway; 3 Clinical Neuroscience, Karolinska Institutet, Stockholm, Sweden

## Abstract

**Abstract:**

It is important to support the Ukrainian mental health research and practice in time of war and after the war has ended. Norwegian universities engage in this by collaboration in psychiatric research and mental health initiatives.

With a grant from the University of Oslo and in collaboration with the Taras Shevchenko National University Kyiv, the Ukrainian Psychiatric Association, and the Norwegian Psychiatric Association, we launched a research school for young psychiatry and psychology researchers who are based in the Ukraine and who can travel outside the country. The research school and training class was held in Warsaw 2023, Krakow 2023 and in Oslo 2024. It offered scientific lectures, interaction with international researchers, and clinical site visits. The young Ukrainian researchers present their projects from early to completed phase to be openly discussed. Networking and scientific exchange is encouraged. The Oslo university library has opened their doctoral educational programs for Ukrainian doctoral students and the University of Oslo sponsors participation in international conferences.

We now aim for a renewed strategy to help meet the needs of mental health and clinical research in the Ukraine also after the end of the war.

**Disclosure of Interest:**

None Declared

